# Commentary: Assessing the Impact and Effectiveness of Hearing Voices Network Self-Help Groups

**DOI:** 10.3389/fpsyg.2017.01856

**Published:** 2017-10-20

**Authors:** Alison Branitsky

**Affiliations:** Department of Psychology and Education, Mount Holyoke College, South Hadley, MA, United States

**Keywords:** hearing voices movement, peer-support, auditory hallucinations, self-help, psychosocial interventions, user-led services

For nearly 30 years, the Hearing Voices Movement (HVM) has been promoting a person-centered, recovery-oriented approach to voice-hearing which frames it as a common, psychologically meaningful human experience that can be explored and understood (Romme and Escher, 1993). The Movement was inspired by the work of social psychiatrist Marius Romme and researcher Sandra Escher who, amongst other research performed in partnership with both patient and non-patient voice hearers, found that many individuals benefit from being able to discuss and make sense of their voices alongside those with similar experiences (Romme and Escher, 1989, 1993, 2000). While Romme and Escher were not the first to suggest a non-pathologizing approach to voice hearing (e.g., De Shazer, 1998, pp. 140–143), they were the first to encourage discussion about the content and origin of their voices in conjunction with other voice hearers.

A direct consequence of this early work was the widespread dissemination of hearing voices self-help groups: service-user directed initiatives in which individuals can explore the content of their voices, exchange coping strategies, and connect with others who have “been there” (Dillon and Hornstein, 2013). In turn, while the HVM emphasizes the well-evidenced link between psychosocial adversity and voice-hearing (Read et al., 2005; Bentall et al., 2012; Varese et al., 2012; Corstens and Longden, 2013), no single framework is privileged over another and individuals are encouraged to explore subjective, personally meaningful frameworks that best describe their experiences (e.g., spiritual, cultural) without having any specific model imposed upon them. Furthermore, an important goal of hearing voices groups is to help individuals foster a positive identity as a voice hearer and to develop more peaceful, constructive relationships with the voices that they hear. As such, voice cessation is not considered the only indication of success as opposed to supporting individuals to relate to and live alongside their voices (Romme et al., 2009).

Since its inception the HVM has established hearing voices groups in over 31 countries in settings ranging from secure forensic units to peer-run community based groups (Dillon and Longden, 2012). However, despite the global dissemination of the approach and the substantial personal testimony attesting to its value (Romme et al., 2009; Woods, 2013; Corstens et al., 2014), little research has been conducted to evaluate the effectiveness of hearing voices groups themselves. Available literature is primarily qualitative, and demonstrates that voice hearers find the groups to be an accepting and supportive environment that foster a sense of agency in recovery from mental health difficulties, wherein connecting with other voice hearers and sharing one's experiences can additionally facilitate different aspects of social recovery (dos Santos and Beavan, 2015; Oakland and Berry, 2015; Beavan et al., 2017; Payne et al., 2017).

The work of Longden et al. complements and extends these findings to quantitatively assess the impact and effectiveness of hearing voices groups from the perspective of their members. To assess the different domains potentially impacted by the groups, the Hearing Voices Group Survey was developed; a 40-item self-report questionnaire designed to assess (1) participants' experiences within the group (e.g., “the group has helped me feel less distressed by my voices,” “I have found it useful to meet other voice hearers in the group”), (2) the social, occupational, and clinical implications of membership outside the group [e.g., “the group has helped me make more friends,” “the group has helped me feel more confident in work,” “because of the group, I have used alcohol (much more often – much less often)”], and (3) the impact of the group on emotional well-being [e.g., “since attending the group, I feel (much more hopeful – much less hopeful)”]. While most of the questions were phrased in the affirmative (e.g., “the group has helped me cope with my voices”), there were several questions that were phrased in the negative (e.g., “the group makes me feel pessimistic about my future”), thereby allowing members to express their concerns with group participation. Items were scored on a five-point Likert-scale, and analyzed by comparing individual item scores to the neutral midpoint. The survey was sent to 62 community-based groups throughout England and a total of 101 responses were returned. Members identified a number of benefits of group attendance, with the provision of support that was unavailable elsewhere and the helpfulness of meeting other voice hearers being amongst the most strongly-endorsed items. Furthermore, positive experiences within the group, such as finding support and having a place to discuss and make sense of voice hearing experiences, were positively associated with both social and emotional outcomes including making more friends and deepening bonds with family, as well as feeling better about oneself, feeling more equipped to cope with distressing voices, and feeling more hopeful about the future. Notably, approximately one-third of participants stated that they found the group distressing at times, yet despite this discomfort still reported that group attendance was beneficial overall. This is understandable given that the majority of participants agreed that the group was “a safe place to discuss difficult things” and implies that, at least for some individuals, the short-term distress of discussing these difficult issues may still be associated with long-term gain.

There were some limitations to the study, including the cross-sectional design, the possibility of a self-selection bias, and the inability to gauge response rate. Nevertheless, it adds to a growing body of evidence of the value of psychosocially-informed approaches to voice hearing, and specifically demonstrates that hearing voices groups can help promote and facilitate recovery through a process of solidarity and shared wisdom that is inherent to peer-support. In turn, the fact that there were no significant differences in reported satisfaction between groups that had only voice hearers as facilitators, only mental health workers as facilitators, or voice hearers and workers co-facilitating indicates that healthcare professionals can use their skills and expertise to support the successful running of such groups.

## Author contributions

The author confirms being the sole contributor of this work and approved it for publication.

### Conflict of interest statement

The author has non-financial ties to the Hearing Voices Network - USA.

## References

[B1] Beavan V. de Jager A. dos Santos B. (2017). Do peer-support groups for voice-hearers work? a small scale study of Hearing Voices Network support groups in Australia. Psychosis 9, 57–66. 10.1080/17522439.2016.1216583

[B2] Bentall R. P. Wickham S. Shevlin M. Varese F. (2012). Do specific early-life adversities lead to specific symptoms of psychosis? a study from the 2007 The Adult Psychiatric Morbidity Survey. Schizophr. Bull. 38, 734–740. 10.1093/schbul/sbs049 22496540 PMC3406525

[B3] Corstens D. Longden E. (2013). The origins of voices: links between life history and voice hearing in a survey of 100 cases. Psychosis 5, 270–285. 10.1080/17522439.2013.816337

[B4] Corstens D. Longden E. McCarthy-Jones S. Waddingham R. Thomas N. (2014). Emerging perspectives from the Hearing Voices Movement: implications for research and practice. Schizophr. Bull. 40(Suppl. 4), S285–S294. 10.1093/schbul/sbu007 24936088 PMC4141309

[B5] De Shazer S. (1998). Clues: Investigating Solutions in Brief Therapy. New York, NY: W. W. Norton.

[B6] Dillon J. Hornstein G. (2013). Hearing voices peer support groups: a powerful alternative for people in distress. Psychosis 5, 286–295. 10.1080/17522439.2013.843020

[B7] Dillon J. Longden E. (2012). Hearing voice groups: creating safe spaces to share taboo experiences, in Psychosis as a Personal Crisis: An Experience-Based Approach, eds M. Romme and S. Escher (London, UK: Routledge), 129–139.

[B8] dos Santos B. Beavan V. (2015). Qualitatively exploring Hearing Voices Network groups. J. Ment. Health Train. Educ. Pract. 10, 26–38. 10.1108/JMHTEP-07-2014-0017

[B9] Oakland L. Berry K. (2015). “Lifting the veil”: a qualitative analysis of experiences in Hearing Voices Network groups. Psychosis 7, 119–129. 10.1080/17522439.2014.937451

[B10] Payne T. Allen J. Lavender T. (2017). Hearing Voices Network groups: experiences of eight voice hearers and the connection to group processes and recovery. Psychosis. 9, 205–215. 10.1080/17522439.2017.1300183

[B11] Read J. van Os J. Morrison A. Ross C. (2005). Childhood trauma, psychosis and schizophrenia: a literature review with theoretical and clinical implications. Acta Psychiatr. Scand. 112, 330–350. 10.1111/j.1600-0447.2005.00634.x 16223421

[B12] Romme M. Escher S. (1989). Hearing voices. Schizophr. Bull. 15, 209–216. 10.1093/schbul/15.2.209 2749184

[B13] Romme M. Escher S. (1993). Accepting Voices. London, UK: Mind Publications.

[B14] Romme M. Escher S. (2000). Making Sense of Voices. London, UK: Mind Publications.

[B15] Romme M. Escher S. Dillon J. Corstens D. Morris M. (2009). Living with Voices: 50 Stories of Recovery. Ross-on-Wye, UK: PCCS.

[B16] Varese F. Smeets F. Drukker M. Lieverse R. Lataster T. Viechtbauer W. (2012). Childhood adversities increase the risk of psychosis: a meta-analysis of patient-controlled, prospective- and cross-sectional cohort studies. Schizophr. Bull. 38, 661–671. 10.1093/schbul/sbs050 22461484 PMC3406538

[B17] Woods A. (2013). The voice-hearer. J. Ment. Health 22, 263–270. 10.3109/09638237.2013.799267 23691942 PMC3836250

